# Supplementary material for: Kinetic and structural mechanism for DNA unwinding by a non-hexameric helicase
Source: Nat Commun. 2021 Dec 1;12:7015. doi: 10.1038/s41467-021-27304-6 (PMC8636605; doi:10.1038/s41467-021-27304-6)
Supplement: Supplementary file 1 — Supplementary Information [file 41467_2021_27304_MOESM1_ESM.pdf]

Supplementary Figures

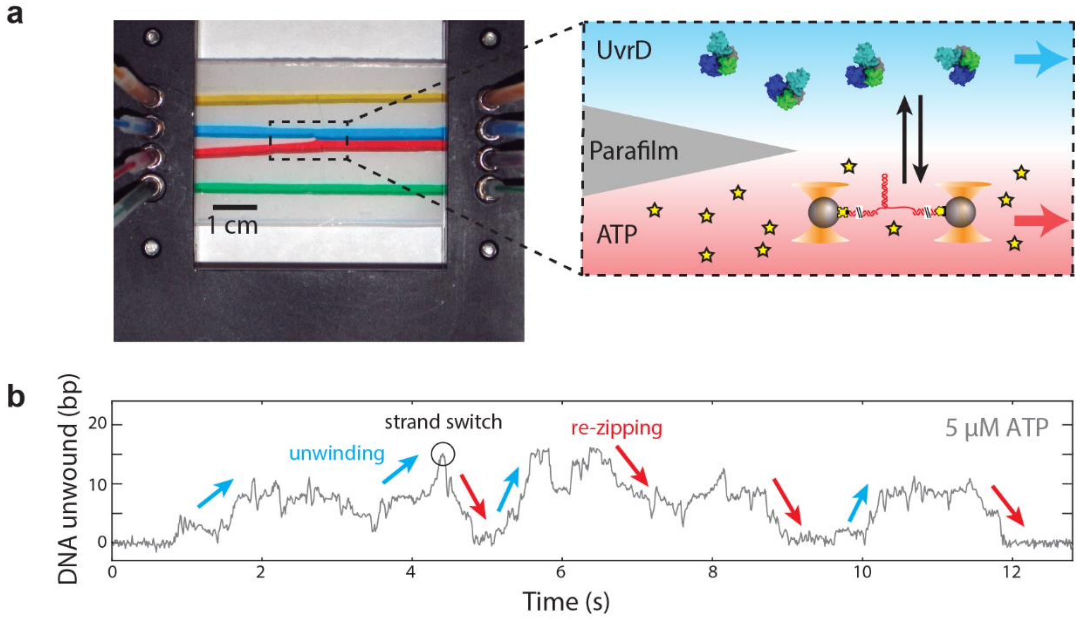

Supplementary Figure 1. Sample chamber design. (**a**) Left: photograph of a laminar flow sample chamber, with different colored food dyes contained in each channel. The polystyrene beads functionalized for tether formation enter the two central channels via thin glass capillaries that connect them to the two outer channels of the chamber (yellow and green in the photograph). Right: Schematic of the two central channels (red and blue in the photograph). Two separate streams containing different buffer components merge at the end of a parafilm taper to form a smooth interface. The top channel contains UvrD but no ATP, while the bottom channel contains ATP but no UvrD. A single DNA hairpin tether is formed in the bottom channel, held at constant force, moved to the top channel to load a UvrD monomer for a 15-30 s incubation period, and finally moved back to the bottom channel to initiate unwinding. (**b**) Example trace of UvrD unwinding activity at a force of 11 pN and 5 µM ATP. Addition of ATP occurs at ~1 s, leading to UvrD-catalyzed unwinding until dissociation of UvrD at ~12 s. Helicase activity is characterized by short, repetitive cycles of unwinding and re-zipping. Source data are provided as a Source Data file.

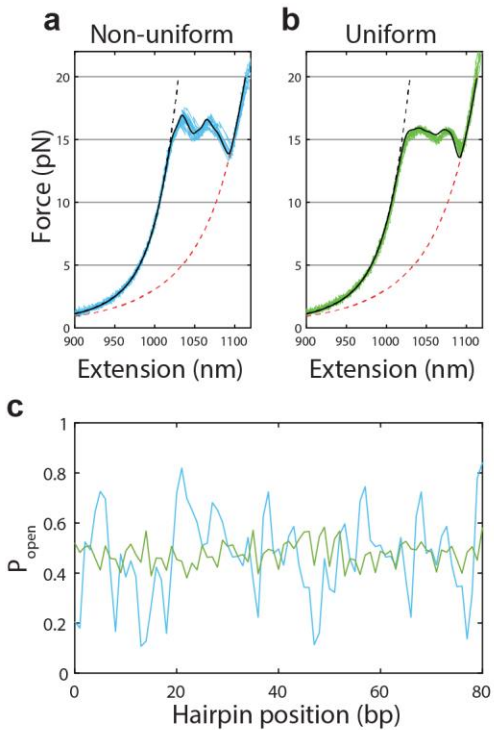

Supplementary Figure 2. DNA hairpin force-extension behavior. (**a**) and (**b**) Representative force-extension curves for hairpin sequences with non-uniform (a, light blue) and uniform (b, green) GC distribution in the hairpin stem. Black and red dotted lines represent fits to the extensible worm-like chain model for the closed and open forms of the hairpin, respectively. The solid black line represents a hairpin unfolding model based on the nearest neighbor base-pairing free energies of the stem sequence (see Methods). N = 12 total force extension curves are shown for each hairpin. (**c**) Plot of P_{open} versus hairpin position at force F = 12 pN, for non-uniform and uniform sequences (same color scheme as above). $P_{open}(F)$ is the thermodynamic probability that one or more base pairs downstream of a certain position will open thermally at force F. Source data are provided as a Source Data file.

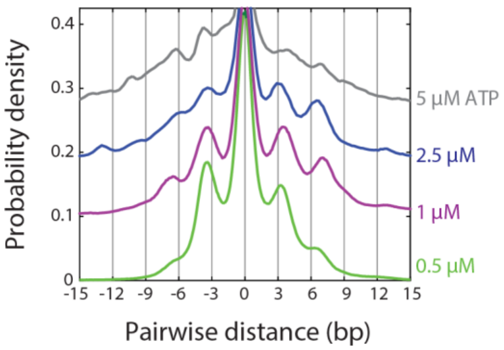

Supplementary Figure 3. Pairwise distance distributions for unwinding and re-zipping. Plots are color coded by individual ATP concentration according to the legend. The same intervals from helicase activity traces that were fitted by the step-finding algorithm were compiled to construct the individual pairwise distance plots at each ATP concentration. We use a signed pairwise distance to account for both unwinding and re-zipping. Data is not shown for 10 µM ATP because high noise and small dwell times make it difficult to discern clear peaks in the pairwise distance distribution at that concentration. The total number of traces contributing to the pairwise distributions are 25, 16, 12, 12 for [ATP] = 0.5, 1, 2.5, 5 µM, respectively. Source data are provided as a Source Data file.

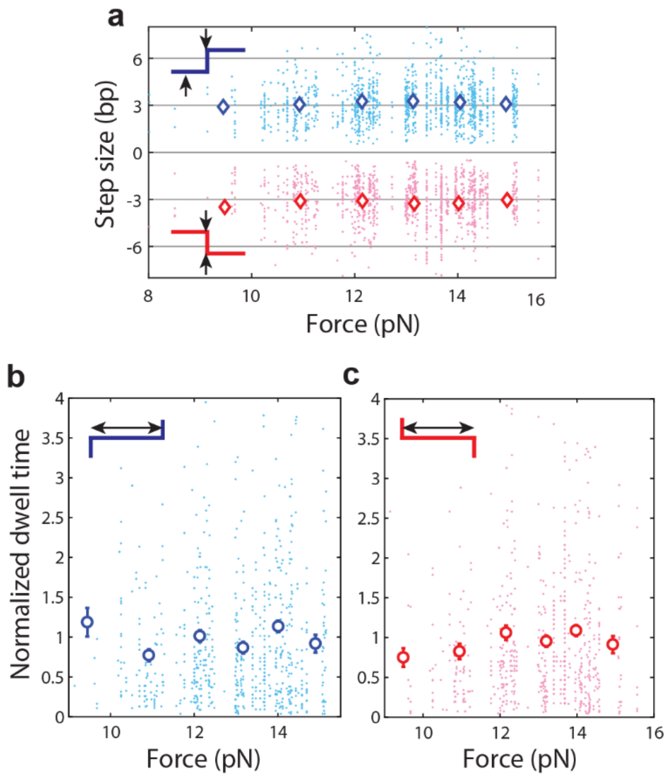

Supplementary Figure 4. Step sizes and dwell times are independent of applied force. (a) Scatter plot of individual unwinding (blue dots; $N = 1206$) and re-zipping (pink dots; $N = 1161$) step size measurements versus applied force, pooled across all ATP concentrations. Averages (colored diamonds) were calculated by binning the data in 1-pN increments across the applied force range, with the exception of data at forces ≤10 pN which were grouped into a single bin due to the smaller number of data points. The s.e.m. is smaller than the diamond symbol size. **(b)** and **(c)** Scatter plots of individual +/+ (blue dots, b; $N = 720$) and -/- (pink dots, c; $N = 671$) dwell times versus applied force, combined for all ATP, with the individual dwell times at each ATP normalized by the average dwell time at that ATP concentration. Averages (colored circles) of these normalized dwells were calculated over the same 1-pN bins used for the step size data. Error bars represent the s.e.m. Steps and dwells obtained from 77 traces across ATP. Source data are provided as a Source Data file.

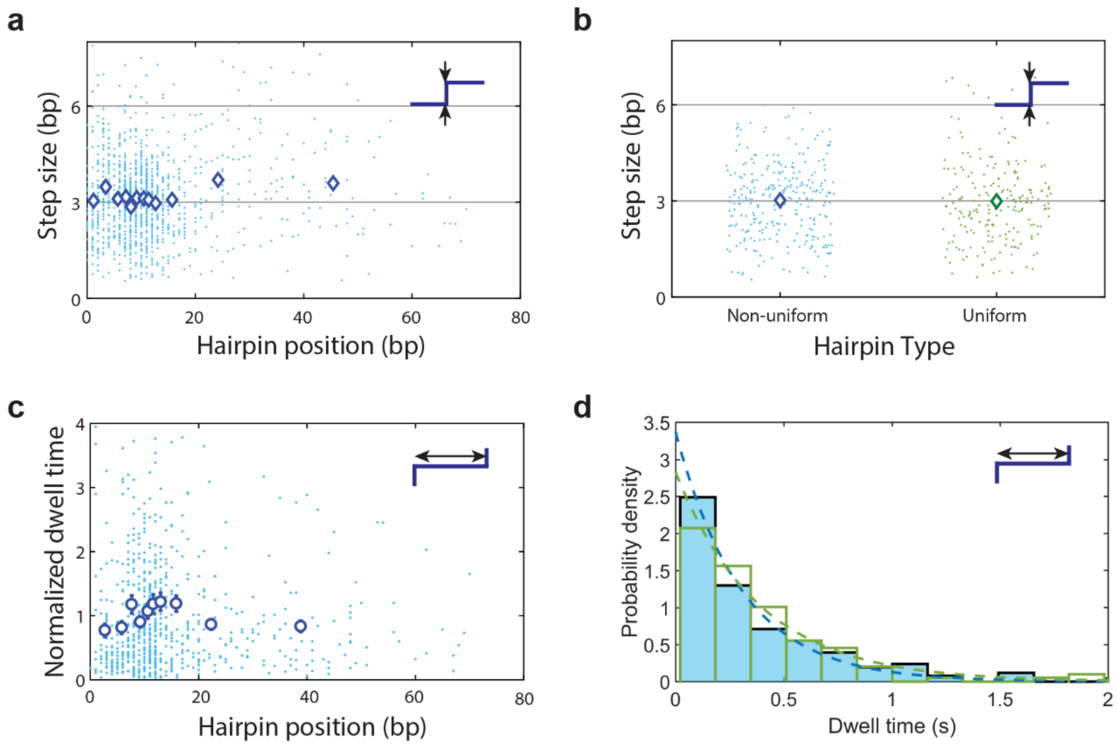

Supplementary Figure 5. Step sizes and dwell times are independent of DNA sequence. (a) and **(c)** Scatter plots of individual unwinding step size (blue dots, a; N = 1206) and +/+ dwell time (blue dots, c; N = 720) measurements pooled across all ATP versus helicase position along the hairpin sequence. The plots show boxcar averages over 100 data points for the step size (blue diamonds) and 70 data points for the dwell times (blue circles). +/+ dwell times combined across ATP are normalized before boxcar averaging in the same manner as for Supplementary Figure 4. Steps and dwells obtained from 77 traces across ATP. **(b)** Comparison of unwinding step size on two hairpin sequences (see Supplementary Figure 2). Scatter plot of individual step size measurements for the non-uniform (blue dots; N = 287) and uniform sequences (green dots; N = 211) at 1 μM ATP, with averages (colored diamonds). Throughout, error bars denote s.e.m., which is smaller than the diamond symbol size in the step size plots. **(d)** Comparison of dwell time distributions between the non-uniform (light blue bars) and uniform sequences (green bars) for +/+ step pairs at 1 μM ATP. The dotted lines are fits to a single-exponential function. Total number of +/+ dwells in the distributions: N = 156 (non-uniform) and 123 (uniform), from 16 and 10 traces, respectively. Source data are provided as a Source Data file.

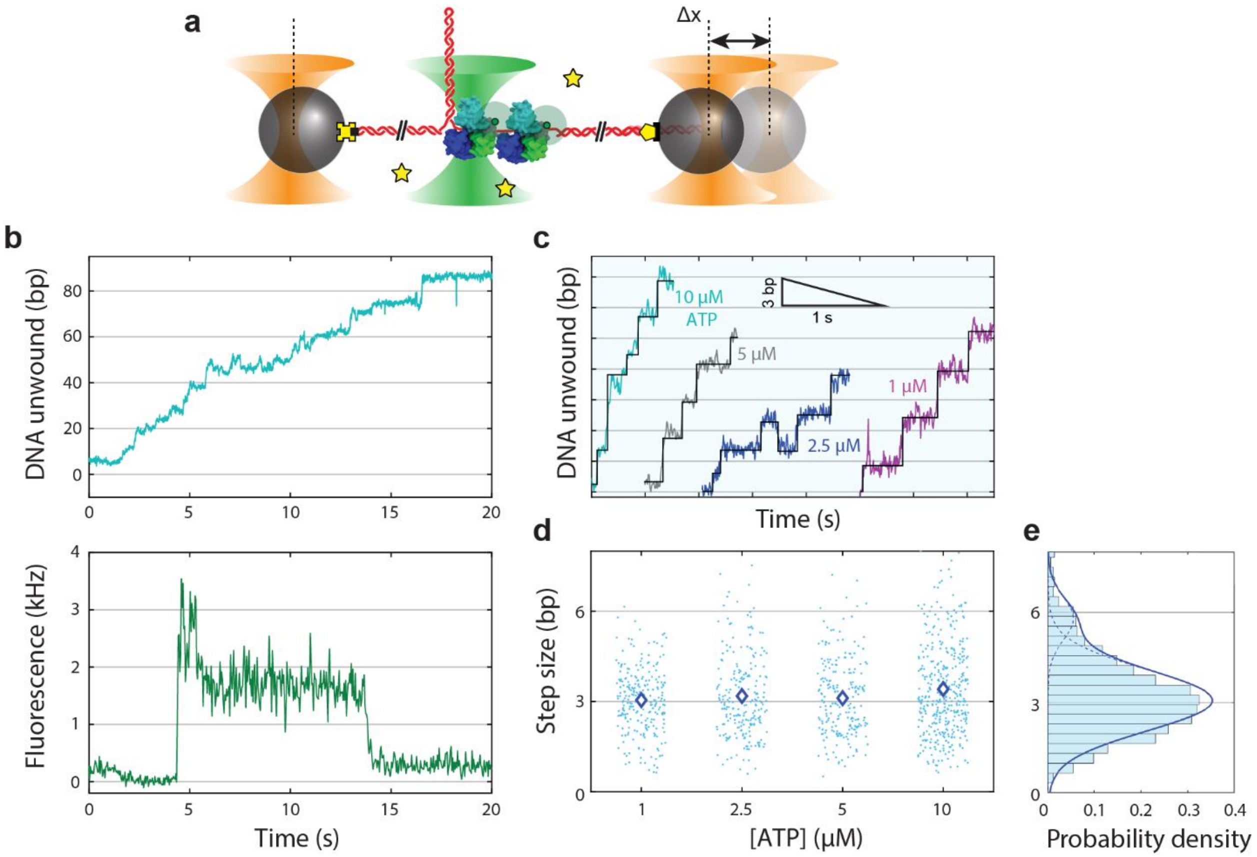

Supplementary Figure 6. Dimeric UvrD unwinds in steps averaging 3 bp. (**a**) Schematic of optical trapping assay with simultaneous confocal fluorescence detection. A DNA hairpin tethered between two optically trapped polystyrene beads contains a 38-dT loading site to allow multiple fluorescently labelled UvrD to bind. Unwinding is detected from a change in tether extension, Δ*x*, at constant force (measured by the optical traps, yellow cones), simultaneously with the number of labeled helicases bound to DNA from fluorescence intensity (measured by the confocal microscope, green cone). (**b**) Example trace at a force of 9 pN and 10 μM ATP showing simultaneous hairpin unwinding signal and fluorescence signal displaying two photobleaching events, indicating the binding of a dimer of UvrD. (**c**) Representative data traces of dimeric UvrD stepping behavior during unwinding across ATP concentrations (color coded as shown) and forces (10-14 pN). (**d**) Scatter plot of individual unwinding step size measurements at each ATP concentration (blue points), and plot of the average step size (blue diamonds). Error bars denoting the s.e.m. are smaller than the diamond symbol size. Total number of unwinding steps displayed: N = 246, 236, 227, 407 from 9, 14, 9, 14 traces at [ATP] = 1, 2.5, 5, 10 μM, respectively. (**e**) Histogram of unwinding (blue) step sizes over all ATP concentrations, with fits to a double-Gaussian distribution (solid lines; dotted lines represent individual single-Gaussian components). Total number of unwinding steps in histogram: N = 1116 from 46 traces across all ATP. Source data are provided as a Source Data file.

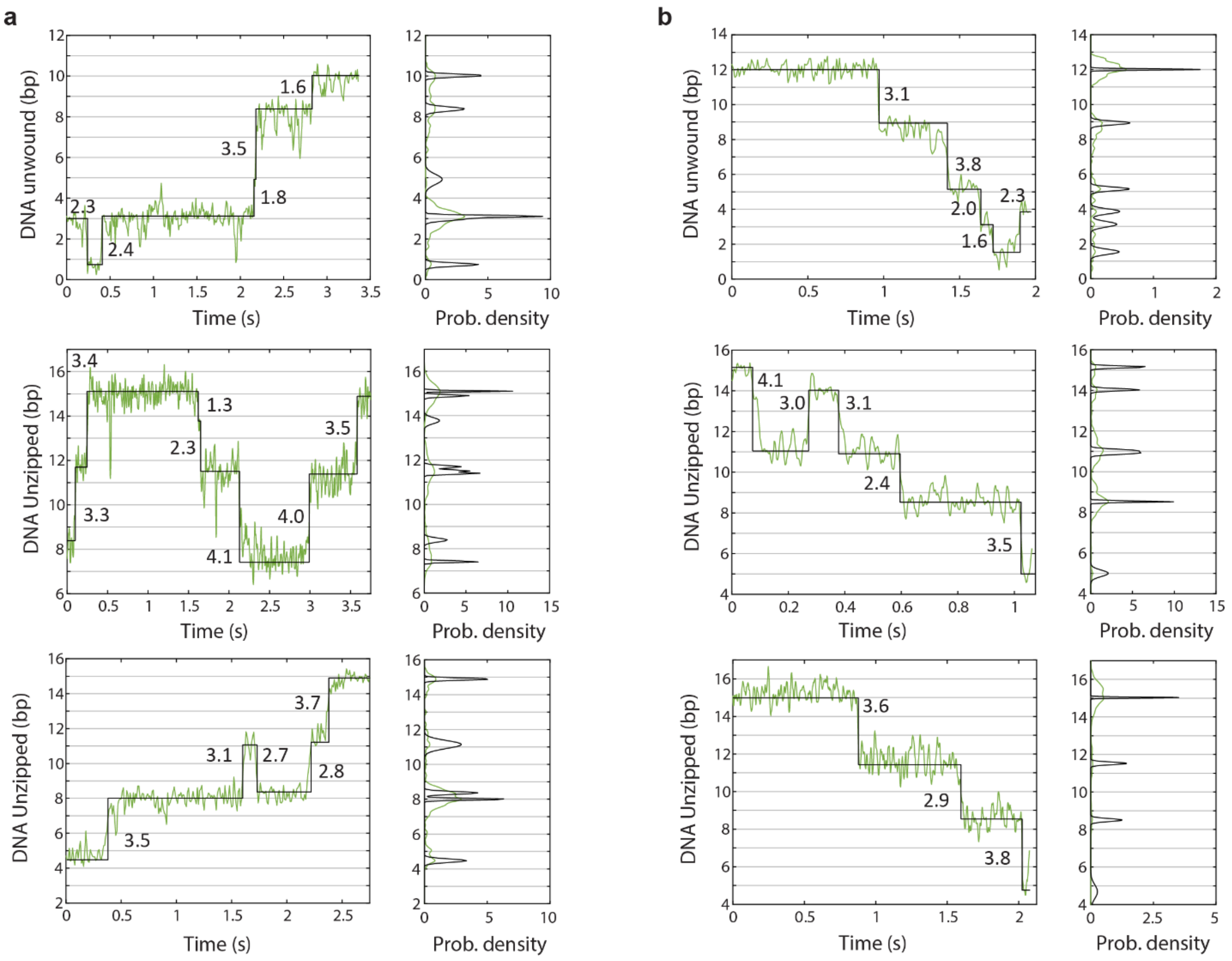

Supplementary Figure 7. Gallery of fitted steps. (**a**) and (**b**) Additional example data traces of unwinding (a) and re-zipping (b), highlighting the prevalence of non-integer steps and steps that differ from the 3-bp average. Data traces at 0.5 µM ATP and force range of 13-15 pN (green; left panels) and corresponding dwell position distributions (green; right panels) are displayed, with fits to steps (black; left panels) and corresponding Gaussian kernels at the most likely dwell position (black; right panels). Individual step sizes in base pairs are indicated on the plots (left panels). Source data are provided as a Source Data file.

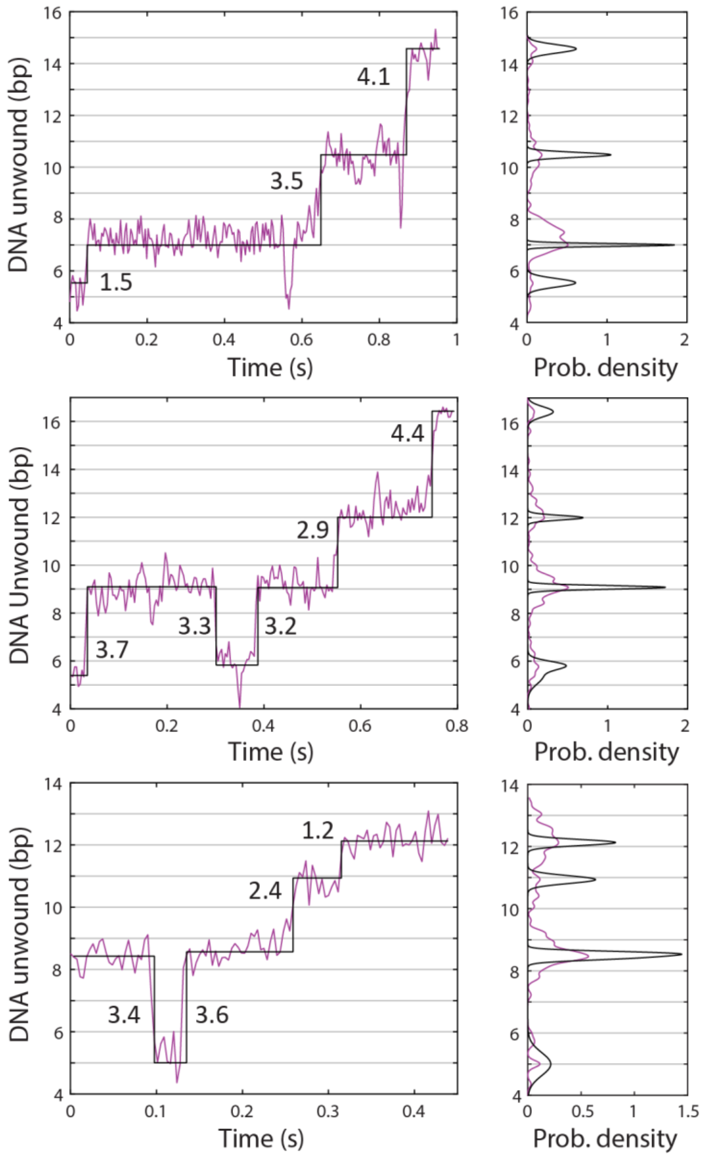

Supplementary Figure 8. Dimeric UvrD exhibits a variable step size and frequent non-integer steps. Representative traces of dimeric UvrD unwinding that emphasize the prevalence of non-integer steps and steps that differ from the 3 bp average. Example data traces at 11 pN and 1 μM ATP (purple; left panels) and corresponding dwell position distribution (purple; right panels), with fits to steps (black; left panels) and corresponding Gaussian kernels at the most likely dwell positions (black; right panels). Individual step sizes in base pairs are indicated on the plots (left panels). Source data are provided as a Source Data file.

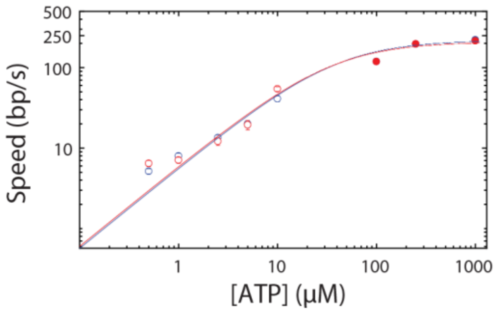

Supplementary Figure 9. ATP dependence of UvrD monomer unwinding and re-zipping speeds. Unwinding (blue) and re-zipping speeds (red) versus ATP concentration for monomeric UvrD, with fits to Michaelis-Menten kinetics (blue and red lines). Data collected over the force range 9-15 pN. At low ATP concentrations (0.5-10 µM, open circles), speeds were calculated by dividing the average step size by the average +/+ or -/- dwell at each ATP, while at high concentrations (100-1000 µM, filled circles), speeds were estimated by fitting segments of uninterrupted unwinding or re-zipping to a straight line. Error bars are s.e.m. and in most instances are smaller than the symbol size. Total number of steps used to determine low-ATP speeds: *N* = 394, 287, 191, 134, 200 (unwinding) and 338, 256, 192, 138, 237 (re-zipping) from 25, 16, 12, 12, 12 traces at [ATP] = 0.5, 1, 2.5, 5, 10 µM, respectively. Total number of independent high-ATP speed measurements: 63, 338, 338 (unwinding) and 60, 333, 350 (re-zipping) from 6, 21, 20 traces at [ATP] = 100, 250, 1000 µM, respectively. Source data are provided as a Source Data file.

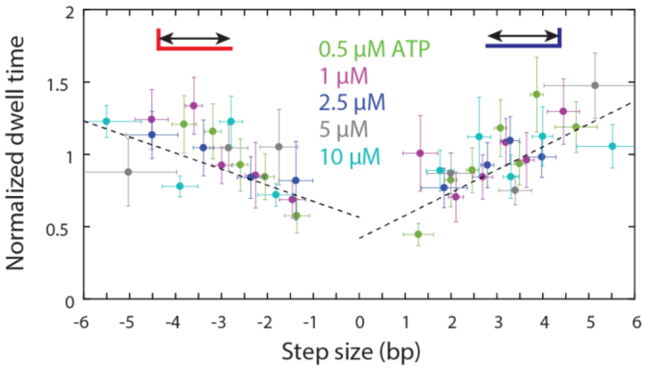

Supplementary Figure 10. Normalized dwell times versus step size. Alternate plot of average dwell time versus step size in Figure 4a, in which the individual +/+ and -/- dwell times at each ATP concentration are normalized by the mean dwell time at that concentration before boxcar averaging over step size. Dotted lines represent fits to linear trendlines as described in the text. Vertical error bars denote standard errors and represent the best estimate of the error on mean normalized dwell time. Horizontal error bars denote standard deviation and represent the spread in the measured step size. Total number of dwells contributing to plots: N = 214, 156, 113, 84, 153 (+/+) and 162, 128, 105, 83, 193 (-/-) from 25, 16, 12, 12, 12 traces at [ATP] = 0.5, 1, 2.5, 5, 10 μM, respectively. Source data are provided as a Source Data file.

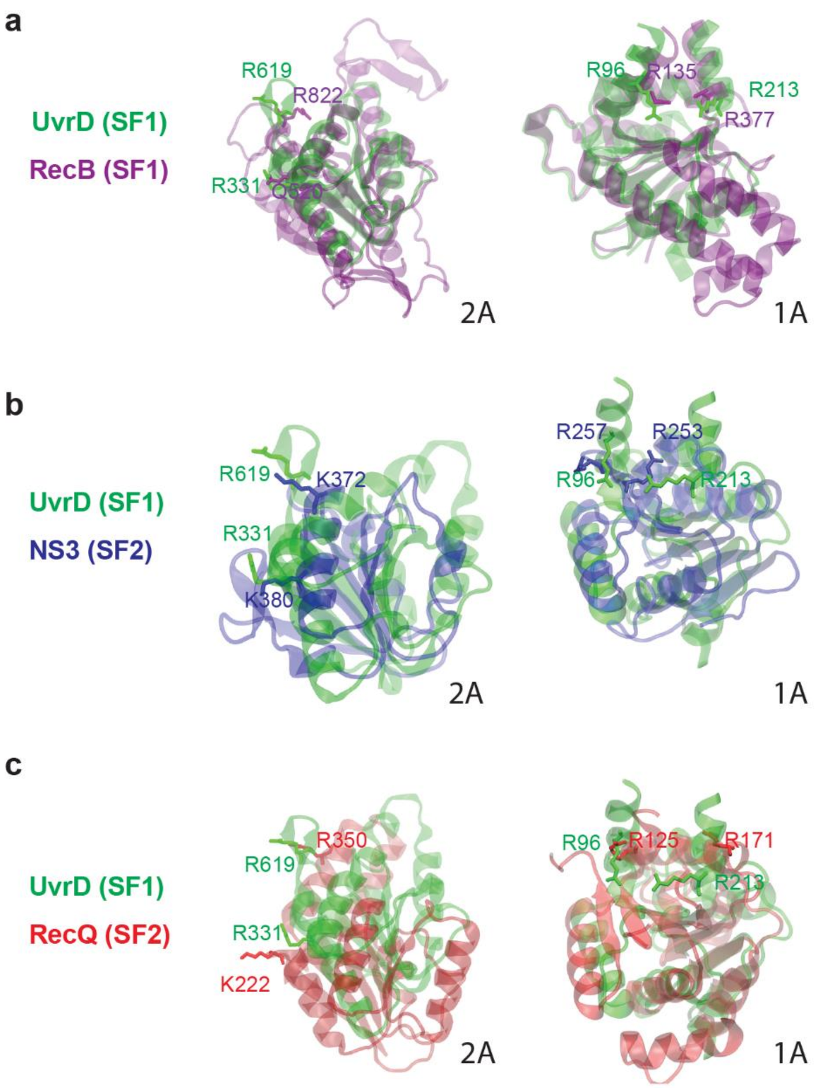

Supplementary Figure 11. Structural alignment of motor core subdomains of other SF1 and SF2 helicases. (**a**) Structural alignment of the 1A and 2A subdomains of UvrD (green) and the RecB subunit of SF1 RecBCD helicase (purple). (**b**) Structural alignment of the 1A and 2A subdomains of UvrD (green) and SF2 helicase NS3 (blue). (**c**) Structural alignment of the 1A and 2A subdomains of UvrD (green) and SF2 helicase RecQ (red). Key UvrD residues that participate in loop formation include R619, R331 in the 2A subdomain (left) and R213, R96 in the 1A subdomain (right) and are well preserved in RecB. For NS3 and RecQ, positively charged arginine or lysine residues are found near the locations of those loop-forming residues in UvrD. Residues for each helicase are colored according to the corresponding structure.

Supplementary Tables

**Oligonucleotide function**	**Hairpin identity**	**Sequence (5'-3')**
Left handle forward primer	Non-uniform	/5Biosg/ TGA AGT GGT GGC CTA ACT ACG
Left handle reverse primer	Non-uniform	CAA GCC TAT GCC TAC AGC AT
Right handle forward primer N-dT	Non-uniform	5Phos/ TTG AAA TAC CGA CCG CTC AGC TAT CAG CC $(dT)_N$ /idSp/ C TCT GAC ACA TGC AGC TCC C
Right handle reverse primer	Non-uniform	/5DigN/CAA CAA CGT TGC GCA AAC T
Hairpin insert	Non-uniform	5Phos/CCT GGG GCT GAT AGC TGA GCG GTC GGT ATT TCA AAA GTC AAC GTA CTG ATC ACG CTG GAT CCT AGA GTC AAC GTA CTG ATC ACG CTG GAT CCT ATT TTT AGG ATC CAG CGT GAT CAG TAC GTT GAC TCT AGG ATC CAG CGT GAT CAG TAC GTT GAC TT
Left handle forward primer	Uniform	/5Biosg/ TGA AGT GGT GGC CTA ACT ACG
Left handle reverse primer	Uniform	CAA GCC TAT GCC TAC AGC AT
Right handle forward primer	Uniform	/5Phos/GA CTG TGA CTG ACA TGA GTG ACT GAG ACT TTT TTT TTT T/idSp/CT CTG ACA CAT GCA GCT CCC
Right handle reverse primer	Uniform	/5DigN/CAA CAA CGT TGC GCA AAC T
Hairpin insert	Uniform	/5Phos/CCT GGA GTC TCA GTC ACT CAT GTC AGT CAC AGT CAG AGT CAT GTC TGA GTC TTG ATG ATG TCA CTG ACT GAG ACT CTG ACT CAC TGA GTC GAG CTT TTG CTC GAC TCA GTG AGT CAG AGT CTC AGT CAG TGA CAT CAT CAA GAC TCA GAC ATG ACT CT

Supplementary Table 1. List of sequences for all hairpin inserts and primers. Abbreviations correspond to the following chemical modifications (readily available from IDT): Phos = phosphate, 5DigN = digoxigenin, Biosg = biotin, idSp = abasic site. For the N-dT loading site, N is either 10, 19, 38, or 60. Note that in the hairpin synthesis, the hairpin insert for the non-uniform sequence is the same regardless of the loading site size in the final construct, as the poly-dT loading site is incorporated into the right handle forward primer.

Sequence	NU	NU	NU	NU	NU	U
[ATP] (µM)	0.5	1	2.5	5	10	1
No. of traces	25	16	12	12	12	10
No. of step fitting intervals	142	91	65	50	58	60
Total No. of unwinding steps	394	287	191	134	200	211
Total No. of re-zipping steps	338	256	192	138	237	154
Unwinding step size (bp)	2.98 ± 0.06	3.02 ± 0.07	3.33 ± 0.10	3.27 ± 0.13	3.62 ± 0.11	2.99 ± 0.09
Re-zipping step size (bp)	2.90 ± 0.06	3.04 ± 0.08	3.11 ± 0.11	3.22 ± 0.13	3.82 ± 0.13	2.99 ± 0.09
Total No. of step pairs	590	452	318	222	379	305
Total No. of +/+ dwells	214	156	113	84	153	123
Total No. of -/- dwells	162	128	105	83	193	71
Mean +/+ dwell time (s)	0.53 ± 0.04	0.37 ± 0.03	0.23 ± 0.02	0.16 ± 0.02	0.08 ± 0.01	0.38 ± 0.04
Mean -/- dwell time (s)	0.42 ± 0.03	0.41 ± 0.03	0.24 ± 0.03	0.16 ± 0.02	0.06 ± 0.004	0.35 ± 0.04

Supplementary Table 2. Key statistics for step size and dwell time data. All errors are standard errors of the mean (s.e.m.). Abbreviations: NU = non-uniform sequence; U = uniform sequence.

[ATP] (µM)	1	2.5	5	10
No. of traces	9	14	9	14
No. of step fitting intervals	100	85	79	147
Total No. of unwinding steps	246	236	227	407
Unwinding step size (bp)	3.05 ± 0.07	3.18 ± 0.09	3.11 ± 0.08	3.41 ± 0.08

Supplementary Table 3. Key statistics for step size data for dimeric UvrD. All errors are standard errors of the mean (s.e.m.).

[ATP] (μM)	100	250	1000
No. of traces	6	21	20
Total No. of unwinding speed fits	63	338	338
Total No. of re-zipping speed fits	60	333	350
Unwinding speed (bp/s)	119 ± 6	196 ± 4	221 ± 5
Re-zipping speed (bp/s)	119 ± 6	197 ± 5	215 ± 7

Supplementary Table 4. Key statistics for velocity data at high ATP for monomeric UvrD. All errors are standard errors of the mean (s.e.m.).
