# Supplementary material for: Kinetic and structural mechanism for DNA unwinding by a non-hexameric helicase
Source: Nat Commun. 2021 Dec 1;12:7015. doi: 10.1038/s41467-021-27304-6 (PMC8636605; doi:10.1038/s41467-021-27304-6)
Supplement: Supplementary file 3 — Description of Additional Supplementary Files [file 41467_2021_27304_MOESM3_ESM.pdf]

Description of Additional Supplementary Files

File name: Supplementary Data 1

Description: Zipped folder containing key intermediate structures from the molecular dynamics simulations as well as essential input and setup files for the simulations.
