# Supplementary material for: Kinetic and structural mechanism for DNA unwinding by a non-hexameric helicase
Source: Nat Commun. 2021 Dec 1;12:7015. doi: 10.1038/s41467-021-27304-6 (PMC8636605; doi:10.1038/s41467-021-27304-6)
Supplement: Supplementary file 5 — Reporting Summary [file 41467_2021_27304_MOESM5_ESM.pdf]

nature research

Corresponding author(s): Yann Chemla

Last updated by author(s): Oct. 22, 2021

Reporting Summary

Nature Research wishes to improve the reproducibility of the work that we publish. This form provides structure for consistency and transparency in reporting. For further information on Nature Research policies, see our Editorial Policies and the Editorial Policy Checklist.

Statistics

For all statistical analyses, confirm that the following items are present in the figure legend, table legend, main text, or Methods section.

n/a	Confirmed	
☐	☒	The exact sample size (n) for each experimental group/condition, given as a discrete number and unit of measurement
☐	☒	A statement on whether measurements were taken from distinct samples or whether the same sample was measured repeatedly
☒	☐	The statistical test(s) used AND whether they are one- or two-sided<br>*Only common tests should be described solely by name; describe more complex techniques in the Methods section.*
☒	☐	A description of all covariates tested
☒	☐	A description of any assumptions or corrections, such as tests of normality and adjustment for multiple comparisons
☐	☒	A full description of the statistical parameters including central tendency (e.g. means) or other basic estimates (e.g. regression coefficient) AND variation (e.g. standard deviation) or associated estimates of uncertainty (e.g. confidence intervals)
☒	☐	For null hypothesis testing, the test statistic (e.g. F, t, r) with confidence intervals, effect sizes, degrees of freedom and P value noted<br>*Give P values as exact values whenever suitable.*
☒	☐	For Bayesian analysis, information on the choice of priors and Markov chain Monte Carlo settings
☒	☐	For hierarchical and complex designs, identification of the appropriate level for tests and full reporting of outcomes
☒	☐	Estimates of effect sizes (e.g. Cohen's d, Pearson's r), indicating how they were calculated

Our web collection on statistics for biologists contains articles on many of the points above.

Software and code

Policy information about availability of computer code

Data collection	Experimental data were collected from a custom-built optical tweezers instrument operated with custom LabVIEW (version 2016) code. The LabVIEW code used in the study is publicly available at: (https://gitlab.com/chemla-lab-public-code/old-trap-labview-code) and (https://gitlab.com/chemla-lab-public-code/fleezer-labview-code). GPU accelerated NAMD2.13 (http://www.ks.uiuc.edu/Research/namd/) was used to perform the molecular dynamics simulations. All the simulation algorithms mentioned in Methods are available options in NAMD.
Data analysis	Analysis of all the experimental data was carried out using custom MATLAB (version R2016b) code, which is publicly available at: https://gitlab.com/chemla-lab-public-code/2021_natcomm_uvrd_stepping_matlab_codes. VMD1.9.4 was used to visualize molecular structures, analyze simulation results, and render images. The MultiSeq available in VMD was used for structural and sequence alignment. The Boxshade program was used to visualize sequence alignment.

For manuscripts utilizing custom algorithms or software that are central to the research but not yet described in published literature, software must be made available to editors and reviewers. We strongly encourage code deposition in a community repository (e.g. GitHub). See the Nature Research guidelines for submitting code & software for further information.

Data

Policy information about availability of data

All manuscripts must include a data availability statement. This statement should provide the following information, where applicable:

- Accession codes, unique identifiers, or web links for publicly available datasets
- A list of figures that have associated raw data
- A description of any restrictions on data availability

Source data for Figures 1-4 and Supplementary Figures 1-10 can be found in the Illinois Data Bank public repository at: https://doi.org/10.13012/B2IDB-5556865_V1. Intermediate structures from the simulations (Fig. 3b), as well as essential input and setup files needed to run the REST2 simulations in NAMD

are provided in Supplementary Data 1. PDB 2IS2 was used to build the initial structure of the UvrD complex. The PDB accession codes for the other helicases whose structures were aligned to that of UvrD are as follows: 1UAA (Rep), 3PJR (PcrA), 5LD2 (RecBCD), 1OYW (RecQ), 1A1V (NS3)

Field-specific reporting

Please select the one below that is the best fit for your research. If you are not sure, read the appropriate sections before making your selection.

☒ Life sciences ☐ Behavioural & social sciences ☐ Ecological, evolutionary & environmental sciences

For a reference copy of the document with all sections, see nature.com/documents/nr-reporting-summary-flat.pdf

Life sciences study design

All studies must disclose on these points even when the disclosure is negative.

Sample size	The sample sizes for all experimental single-molecule data sets, which comprise the entirety of the experimental data, were not computed a priori, but were selected to allow statistically significant conclusions. For our REST2 MD simulations, multiple (20) replicas of the system were simulated simultaneously, although only a single molecule was included in the system. An accumulated 7 microsecond simulation time for each system is considered to be sufficient MD sampling in this case.
Data exclusions	Two individual experimental data points, specifically from the unwinding dwell time data at 2.5 μM ATP and the re-zipping dwell time data at 1 μM ATP, were excluded from analysis by statistical (bootstrap) testing. As described in detail in the Methods section, the two outlier dwell times were removed after being identified as the single data points that made the respective distribution of bootstrapped nmin values multi-modal.
Replication	The number of independent replicates for each single-molecule experiment, defined as the number of helicase unwinding activity traces, are detailed both in Supplementary Tables 2-4 and in the figure legends. As stated above, the single-molecule data sets constitute the entirety of the experimental data. The REST2 molecular dynamics simulations were performed with 20 replicas, each of which lasted 350 ns. In total, a 7 microsecond simulation time was obtained for each system. All attempts at replication were successful.
Randomization	Allocating experimental groups was not relevant for this study because the protein and DNA molecules examined were identical across all replicates for each experiment.
Blinding	Blinding was not relevant to this study because the protein and DNA molecules examined were identical across all replicates for each experiment and for the simulations. Furthermore, analyses were not subjective enough to require researcher blinding.

Reporting for specific materials, systems and methods

We require information from authors about some types of materials, experimental systems and methods used in many studies. Here, indicate whether each material, system or method listed is relevant to your study. If you are not sure if a list item applies to your research, read the appropriate section before selecting a response.

Materials & experimental systems

n/a	Involved in the study	
☒	☐	Antibodies
☒	☐	Eukaryotic cell lines
☒	☐	Palaeontology and archaeology
☒	☐	Animals and other organisms
☒	☐	Human research participants
☒	☐	Clinical data
☒	☐	Dual use research of concern

Methods

n/a	Involved in the study	
☒	☐	ChIP-seq
☒	☐	Flow cytometry
☒	☐	MRI-based neuroimaging
